# Examining the integration of refugees into the national health system in Uganda: an analysis using the policy triangle framework

**DOI:** 10.1186/s13031-024-00640-2

**Published:** 2025-01-21

**Authors:** Henry Komakech, Shatha Elnakib, Lama Bou Karroum, Evelyn Nyachwo, Winnie Adoch, Sarah Sali, Godfrey Goddie Okeny, Christopher Garimoi Orach

**Affiliations:** 1 Department of Community Health and Behavioural Science, School of Public Health, College of Health Sciences, Makerere University, Mulago Hill Road, P.O. Box 7072, Kampala, Uganda; 2 International Health Department, Johns Hopkins Bloomberg School of Public Health, 615 N. Wolfe Street, Baltimore, MD 21205 USA; 3 Center for Systematic Reviews on Health Policy and Systems Research (SPARK), American University of Beirut, Beirut, Lebanon; 4 Department of Health Management and Policy, Faculty of Health Sciences, American University of Beirut, Beirut, Lebanon; 5 School of Women and Gender Studies, College of Humanities and Social Sciences, Makerere University, 45 Pool Road, P.O.BOX, 7062 Kampala, Uganda

**Keywords:** Integration, Refugees, Health policy, Policy triangle, National health system, Uganda

## Abstract

**Background:**

Uganda has been confronted with a sustained influx of refugees for decades. This prompted the government to explore opportunities to integrate refugees into local service structures including its national health system. This paper chronicles the history of policies and strategies that have influenced the integration of refugees into the national health system in Uganda and investigates factors that impacted policy evolution and progression.

**Methods:**

We used a case study approach that drew on a document review and key informant interviews with 28 respondents at national and subnational levels. Interviews were analyzed using thematic qualitative analysis and findings were organized using Walt and Gilson’s Policy Triangle Framework. Data from the literature review, media review, and key informant interviews were triangulated.

**Results:**

Uganda’s experience with the integration of refugee’s dates to 1999 when the country first implemented the Self-reliance strategy. Since then, policy learning and evolution have taken place, with policies around integration evolving and improving over time, moving the country towards more effective implementation of integrated health services. A conducive policy environment was key as a set of legislations at national and district-levels and sector-specific plans and budgets that included refugees have provided the foundation for implementation. The integration received support and buy-in from the highest levels of government including the President and the Office of The Prime Minister. This was coupled with deliberate efforts by the government to mainstream refugee response in local plans and budgets, allowing implementation at district and sub-district levels. These factors were pivotal to the implementation of the integration agenda.

**Conclusion:**

Our study highlights the complex, dynamic, evolving, and multifaceted nature of the multisectoral health policy process in the integration of health services in refugee settings. The findings shed light on the importance of collaboration between stakeholders, mobilization of legal and political frameworks to shape the integration of refugee health services into the national health system, and the importance of ensuring that high-level commitments translate to action and development plans at local levels.

**Supplementary Information:**

The online version contains supplementary material available at 10.1186/s13031-024-00640-2.

## Introduction

Sub-Saharan African countries host an estimated 7 million refugees. A refugee is a person who is outside the country of his nationality (or not having a nationality) and is unable or unwilling to avail himself of the protection of that country due to a well-founded fear of being persecuted for reasons of race, religion, nationality, membership in a particular social group or political opinion [1]. Uganda has been hosting refugees since the pre-independence period. The country hosts the largest number of refugees in Africa and the fifth globally [2] after Turkey, Colombia, Germany, and Pakistan. Refugees in Uganda originate mainly from countries in the Easy African region including South Sudan, Rwanda, Burundi, the Democratic Republic of Congo (DRC), Kenya, Somalia, Eritrea, and Ethiopia. The largest refugee population in the country (867,453), are from South Sudan, 46,707 from Burundi, 402,521 from the DRC, and 77,997 from a mix of other countries [3]. The most recent arrivals of refugees include 3053 from South Sudan, 5392 from Sudan in the northern region, 3182 from the DRC in the western region, and 6286 urban refugees in Kampala [4].

Uganda is a developing country located in East Africa governed based on a loose multi-party system with several political players including political parties, and civil society organizations [5]. Since the 1990s, Uganda's public health system has been decentralized to districts [6] structured into three tiers: national referral hospitals, regional referral hospitals, and district hospitals, complemented by health centers. Despite recent progress being in key health outcomes, access to health services is affected by several factors including inadequate infrastructure, limited access to health services in rural areas, and high disease burden (communicable and non-communicable) [7, 8]. The government has prioritized combating HIV/AIDS, malaria, infectious diseases, maternal and child health initiatives, and ongoing efforts to strengthen health service delivery and health financing. The health sector includes several stakeholders including the Ministry of Health (MoH), international organizations such as the World Health Organization, non-governmental organizations (NGOs), the private sector, and various health professionals working towards improving access to health services.

Over the past two decades, there has been increased emphasis on the need to strengthen national health systems to facilitate access to health services for both refugees and nationals aligning with global refugee treaties. Several refugee-hosting countries have started integrating refugees into national health systems [9]. Uganda initiated the concept of self-reliance before recent initiatives advocating for the integration of refugees into national systems emerged. Key among these was the Comprehensive Refugee Response Framework (CRRF). This CRRF framework is an outcome of the New York Declaration on Refugees that was adopted by the UN General Assembly on September 19, 2016 [10]. Following the success, along with the enactment of other strategies, Uganda is regarded as a model for other countries considering the integration of refugees into the national health system [11].

For decades, Uganda has maintained an open-door policy to all refugees and other displaced populations irrespective of their nationality or ethnicity. Refugees residing in the country’s settlements have freedom of movement and the right to employment [12]. The government allocates land to each refugee family in designated settlements. In addition, refugees have access to all social services in the country. This paper traces the historical progression of policies and strategies that have influenced the integration of refugees into the national health system in Uganda. We do so by examining events that have shaped Uganda’s effort to integrate refugees into local service delivery structures. We document the evolution of policies and strategies and the renewed urgency that integration received in 2015 and onwards.

The analysis explores the development of health policies and strategies aimed at integrating refugees into the national health system using the Walt and Gilson Policy Triangle framework. The framework examines health policies through four key elements including context, content, process, and actors [13]. Content encompasses policy objectives, laws, and guidelines. Context refers to the overarching social, economic, political, and cultural conditions that impact policy formulation and implementation. Process refers to the methodologies employed in policies development, and how they are negotiated, implemented, and evaluated. Lastly, the actors refer to individuals, networks, groups, and organizations involved in policy formulation adoption, and implementation.

## Methods

### Study setting

This study was conducted in three refugee hosting districts of Adjumani, Yumbe, and Obongi, in Uganda. The three districts were selected because of the significant population of refugee, diversity of refugee, and varied urban, peri-urban, and rural settings, offering valuable insights for informing policy and practice in refugee health system integration using the policy triangle framework. The three districts host refugees mainly from South Sudan. Currently, there are 1.5 million refugees in the country. The three study districts host an estimated population of 519,829 refugees. These include 208,191 in Adjumani, 190,256 in Yumbe, and 121,382 in Obongi [14]. Refugees in the three districts live in government-designated settlements. Settlements are interspersed among host communities. Social services, including health care, are provided by the Government of Uganda (GoU) and supported by the United Nations High Commission for Refugees (UNHCR) and other implementing partners in these settlements.

### Study design

This was a case study design [15]. The design was chosen as it is suitable for an explorative study of contemporary events in a real-life context and lends itself to an inquiry into the evolution of policies around the integration of refugees into the national health system in Uganda. The study drew on two data collection methods: document review (peer-reviewed articles and grey literature) and key informant interviews with 28 respondents at national and subnational levels. The policy triangle framework by Walt and Gilson informed data analysis [13].

### Database search strategy

We conducted a search of electronic databases including Medline (Ovid), PubMed, and Scopus for peer-reviewed articles. The databases were searched for articles published between January 2013 to December 2020 using a string of keywords related to the study topic. We used search terms including “refugee”, “refugees” and “Uganda” The search strategies of the different databases are found in (Additional File 1). To ensure the comprehensiveness of the search, the reference lists of the identified studies were reviewed for additional articles related to the study objectives. The research results were reviewed and screened by the authors. Articles were included if (1) addressed the issue of refugees and refugee integration in Uganda, (2) policies and strategies related to the integration of refugees into the national health system were discussed, and (3) they were published in English. Data were extracted into a Microsoft Excel spreadsheet.

### Document review

Grey literature was retrieved from the websites of government ministries and local and international NGOs. We reviewed documents examining the content (effectiveness and feasibility), process (consultation, political context, actors, policy gains), context (drive for policy change, capacity, policy problem), and actors (power). The search for grey literature applied the same keywords and inclusion/exclusion strategy used in the academic database. The search was undertaken by the first author in consultation with the third. We searched the websites of the MoH, Ministry of Disaster Preparedness—Department of Refugees, Ministry of Finance Planning and Economic Development, Ministry of Local Government, Ministry of Justice and Constitutional Affairs, The Parliament of Uganda, UNHCR, World Health Organization (WHO), UNICEF, Medical Teams International, Real Medicine Foundation, and Doctors with Africa. The documents included legislation (law articles and governmental decrees), reports, and news media articles. We also reviewed the reference list of relevant documents. Media articles on refugee issues were identified and collected from two national newspapers: The New Vision and The Daily Monitor.

**Fig. 1:**
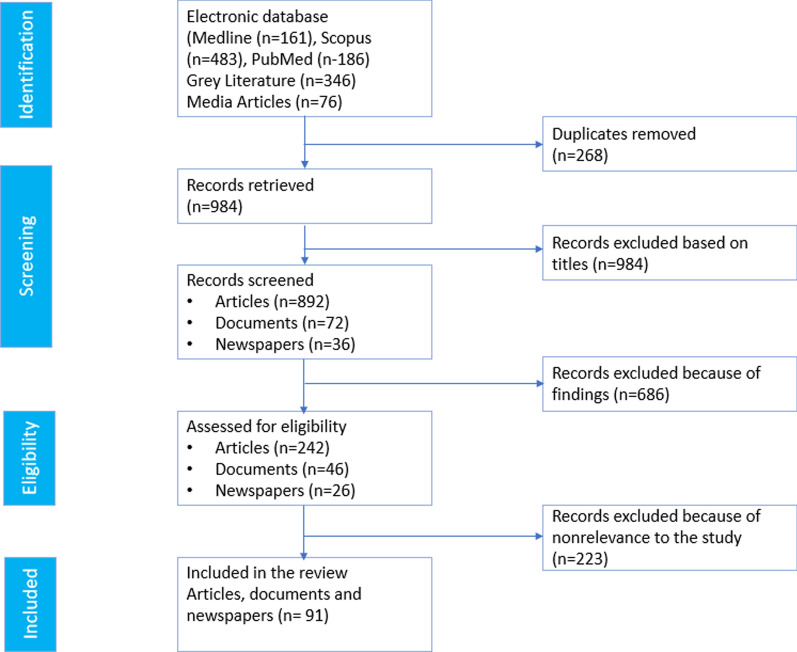
Flow diagram of the selection of articles in the review of the integration of refugee into the national health system in Uganda

In total, 829 peer-reviewed and 72 grey literature documents were retrieved. Of these, 91 documents were eligible for inclusion and review (Fig. 1). The documents were reviewed and summarized in an Excel data collection matrix. The matrix collected information on the title, date of publication, actors, summary/abstract, structural factors, institutional factors, and individual and community level factors relevant to integrations, policies, and financing of health services, among other topics.

### Semi-structured interviews

We conducted semi-structured face-to-face, virtual, and telephone interviews with respondents at national and district levels. We used both purposive and snowball sampling to identify participants with knowledge and expertise in the integration of health services for refugees and host communities in Uganda. We outlined selection criteria for key informants and purposively identified a set of informants who fulfilled the eligibility criteria. We then employed snowball sampling to expand the participant pool, asking our participants for recommendations for additional key informants whose insights would benefit the study (Table 1). The key informants included government officials, civil society, staff of humanitarian agencies, officials of donor organizations, and academics. Specifically, we interviewed three commissioners and two directors of MoH, four personnel from UN organizations, one official of the World Bank, three staff of international NGOs, two university researchers, and five district health officers in refugee-hosting districts. The respondents were knowledgeable and experienced in planning, decision-making, policymaking, and supporting health service delivery to refugees and host communities in the refugee-hosting districts.

**Table 1:** Key informants interviewed for the study

	Gender		Organization	Experience/Involvement		
	Female	Male		10 + years	Level of involvement	Involvement in the policy process
Key informant #1		✓	MoH	✓	National	Policy development
Key informant #2	✓		MoH	✓	National	Policy development and implementation
Key informant #3		✓	MoH	✓	National	Policy development, implementation, and promotion
Key informant #4		✓	MoH	✓	National	Policy development, implementation, and promotion
Key informant #5	✓	✓	MoH	✓	National	Policy development, implementation, and promotion
Key informant #6		✓	The World Bank	✓	National	Policy development and promotion
Key informant #7		✓	Public University	✓	National	Advocacy and research
Key informant #8		✓	Public University	✓	National	Advocacy and research
Key informant #9		✓	WHO	✓	International NGO	Policy development and promotion
Key informant #10		✓	WHO	✓	International NGO	Policy development and promotion
Key informant #11		✓	UNICEF	✓	International NGO	Policy implementation
Key informant #12	✓		UNFPA	✓	International NGO	Policy implementation
Key informant #13	✓		DLG	✓	Sub-national	Policy implementation
Key informant #14		✓	DLG	✓	Sub-national	Policy implementation
Key informant #15		✓	DLG	✓	Sub-national	Policy implementation
Key informant #16	✓		NGO		Sub-national	Policy implementation
Key informant #17		✓	NGO	✓	Sub-national	Policy implementation
Key informant #18		✓	NGO		Sub-national	Policy implementation
Key informant #19	✓		OPM-MoDP	✓	National	Policy implementation
Key informant #20		✓	OPM-MoDP	✓	National	Policy implementation
Key informant #21		✓	OPM-MoDP	✓	National	Policy implementation
Key informant #22		✓	OPM-MoDP	✓	National	Policy development
Key informant #23		✓	OPM-MoDP	✓	National	Policy development
Key informant #24		✓	OPM-MoDP	✓	National	Policy development
Key informant #25		✓	OPM-MoDP	✓	National	Policy implementation
Key informant #26		✓	CAO	✓	Sub-national	Policy implementation
Key informant #27		✓	CAO	✓	Sub-national	Policy implementation
Key informant #28		✓	CAO	✓	Sub-national	Policy implementation

CAO, Chief Administrative Officer; DLG, District Local Government; OPM, Office of the Prime Minister; MoDP, Ministry of Disaster Preparedness; MoH, Ministry of Health; NGO, Non-government Organization

All the interviews were conducted by authors with the assistance of trained research assistants. Interviews were conducted by the moderator and a note taker in a quiet and convenient location with the respondent’s consent. The interviews were conducted using a semi-structured interview guide (Additional File 2). The interview guide contained questions developed based on the study objectives, and literature review. All interviews were digitally recorded and saved after consent was obtained from the respondent. During the interviews, a notetaker took handwritten notes. The interviews lasted between 45 min and one and a half hours. At the end of the day interview, handwritten notes were reviewed by the authors and interviewers. We used an integrative method, including notetaking during interviews and a sampling method ensuring maximum diversity, which provided a means for ensuring data reliability and transferability.

### Data management

Interview data was transcribed and coded using a codebook that was developed a priori based on the study objectives [13]. Initially, we assumed that Kingdon’s multiple streams model offered an appropriate analytical lens and used codes reflecting the three streams to capture important insights. However, it soon became clear from the data that the framework was not suitable for the Ugandan context. Instead, Walt and Gilson's policy triangle framework served as a better fit to document the evolution and iterative progression of policies around integration. After the completion of coding, the authors organized an analysis workshop during which they reviewed coded excerpts and synthesized findings.

### Data analysis

The data from the document review was triangulated with in-depth interviews during the analysis process. This served to provide a deeper understanding of the underlying motivations, attitudes, and decision-making processes that may not be apparent from the use of just one of the methods alone. Through data triangulation and thematic analysis, the integration of findings from both sources enriched the study's depth and breadth, enabling a comprehensive understanding of the development of the policy agenda for the integration of refugees into the health system in Uganda. This contributed to the generation of nuanced and contextually grounded insights.

The data from the documents were analyzed using the content analysis technique. The policy triangle framework guided the analysis process. Excerpts were organized by the Context, actors, content, and processes framework post-hoc [16]. The analysis reviewed the policy content including the subject matter of the policy, objectives, strategies, and guidelines to achieve the objectives. The policy process assessed how the policy came into existence and was implemented [17]. The analysis of actors examined who were the key stakeholders with influential roles and responsibilities in the policy process. In addition, we assessed systemic and structural factors including social, economic, political, and others influencing the integration of refugees into the national health systems.

The thematic analysis utilized a methodology that incorporated both concept-driven and data-driven coding techniques. First, all transcripts from the key informant interviews were read by the authors HK, EN, and SE, and all authors were involved in and contributed to the data analysis. The transcripts were read and reviewed several times and coded thematically. We then coded the transcripts according to predefined codes from the policy analysis framework. In addition, other emerging themes were identified from the data. The policy analysis triangle framework was populated with findings from the study. The authors discussed and interpreted the findings until a joint understanding was achieved. Coding was done using Quirkos software.

The articles and documents were screened by HK and LBK. A random sample of excluded studies was reviewed and checked by CGO. Consensus was gained for uncertain articles through discussion between HK, CGO, and LBK. Included studies were screened based on the title and abstract and the full paper was verified through assessment of its contents based on the inclusion criteria. Data extraction was carried out by HK and LBK. The extracted data were entered in an MS Excel spreadsheet and included variables such as the title of the document, author, language, keywords, factors shaping policies that support the integration of health services (structural, institutional, and individual), health systems arrangements (financing, availability, and quality).

**Fig. 2:**
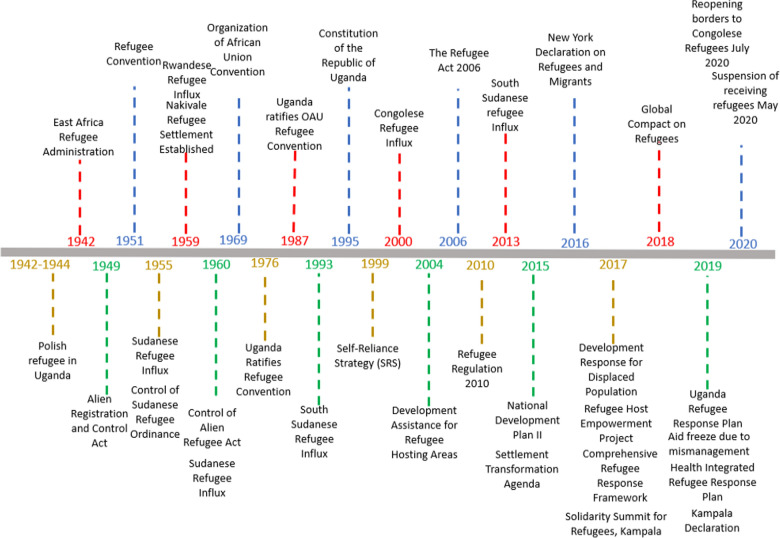
Timeline of key events and strategies in Uganda

Following the completion of the analysis, documents were organized into a timeline based on the date of publication to visualize the evolution of policies and strategies related to the integration of refugees into the national health system in Uganda. The timeline supplemented the document review and enabled visualization of the major refugee events, strategies and policies (Fig. 2).

## Results

### Timeline

**Table 2:** Key events and policies on integration of refugees in Uganda

Date	Event/policy
1942–1944	Polish refugees, mainly women and children, arrived in Uganda during World War (WW) II. The refugees settled in two camps in western Uganda and central Uganda [18]
1955	Sudanese Refugee Influx 1955 as a result of civil unrest in Sudan. Refugees settled in the West Nile region [19]
1959	Nakivale settlement was established in 1959 and the policy of land allocation to refugees was introduced. Rwandese refugees were the first group to settle in the Nakivale settlement [20]
1959–1960	The influx of Rwandese Refugees 1959 First refugee settlements were established in Nakiwale and Oruchinga in western Uganda [20]
1960	Control of Alien Refugees Act (CARA) repealed the Control of Refugees from Sudan Ordinance 1955 and aimed to control Rwandese refugees in Uganda. It was considered a restrictive approach to addressing forced displacement that was “based on control rather than protection,” and therefore granted the authorities “wide discretionary power” [19]
1960	Over 86,000 Sudanese refugees sought refuge in Uganda [21]
1976	Uganda ratified the 1951 Refugee Convention and its 1967 Protocol but with several reservations [22]
1987	Uganda ratified the 1969 Organization of African Union [23] Convention on Refugees Some of the provisions in the CARA, such as the tight restrictions on refugees’ freedom of movement, contradicted Uganda’s obligations under these conventions [24]. However, the CARA was not fully implemented: the government applied it “mostly to situations of mass influx,” but implemented “practices in respect of individual refugees that were at least partly informed by Uganda’s regional and international obligations [25]
1993–1994	Over 200,000 South Sudanese arrived in the West Nile region and settled in Koboko then Arua, Moyo, and Adjumani districts [26]
1995	The Constitution of the Republic of Uganda provides several rights to refugees, but it quietly excludes refugees from becoming citizens, whether by birth or by registration [27]
1999	The Government of Uganda developed and started implementing the Self-Reliance Strategy (SRS) for refugees which aimed to improve food self-sufficiency, harmonize social services delivery, and support local government capacity in essential services delivery [28]
2000	Over 12,000 Congolese refugees fled to Uganda from the Democratic Republic of Congo and settled in Nakivale and Oruchinga settlements in western Uganda [29]
2004	Development Assistance to Refugee (DAR)-Hosting Areas program that aimed to strengthen food self-sufficiency, harmonize social services delivery and support local government capacity in essential services delivery [30]
2006	Uganda enacted the Refugee Act 2006 replacing replaced the Control of Aliens Act 1960 and provided several provisions for the management of refugees in Uganda [12]
2010	Uganda passed its Refugee Regulations in 2010 incorporating international obligations into domestic law to reinforce the provisions of the Refugee Act 2006 [12]
2013	The influx of South Sudanese refugees as a result of the escalation of civil war between Sudan’s Peoples Liberation Movement factions and government forces (SPLM/A) [29]
2014	The Development Response to Displaced Populations (DRDIP) project aims to improve access to social services, expand economic opportunities, and enhance environmental management for both refugees and host communities [31]
2015	The integration of refugees into the National Development Plan II (NDPII, 2015/16–2019/20). The Settlement Transformation Agenda (STA) was included in the NDP II therefore making refugees part and partial of the development agenda in Uganda [32]
2016	The settlement Transformation Agenda (STA) emphasizes a non-encampment policy for refugee protection and assistance. Refugees are provided a plot of land for housing and cultivation alongside host communities [33]
2016	Uganda signed the New York Declaration in 2016. The declaration reaffirmed the importance of the international refugee protection regime, committed fully to respect the rights of refugees and migrants, pledged to provide more predictable and sustainable support to refugees and host communities, and agreed to expand opportunities to achieve durable solutions [34]
2017	Refugee and Host Population Empowerment (ReHoPE) is an approach to deliver protection, and social and economic development in refugee hosting areas in Uganda to ensure that both refugees and host communities are served equally in Uganda. ReHoPE forms a critical component of Pillar Three of the Ugandan Comprehensive Refugee Response Framework (CRRF) model with its focus on resilience and self-reliance [35]
2017	The Solidarity Summit in Uganda aimed to galvanize support and strengthen Uganda’s progressive and transformative approach to refugee protection and assistance [36]
2017	Uganda launched and adopted the Comprehensive Refugee Response Framework (CRRF) to ensure access to territory and the principle of nonrefoulement, provision of individual registration and documentation, access to social services including education and health, the right to work, and the right to establish business [10]
2018	Uganda Global Compact on Refugees adopted [37]
2019	Uganda Country Refugee Response Plan (UCRRP) contributes to achieving the CRRF in Uganda, alongside interventions carried out by government institutions [38]
Feb 2019	Germany, Japan, and the UK’s Department for International Development (DFID) suspended funding to UNHCR Uganda due to mismanagement of funds between 2016 and 2017 affecting protection and assistance and other development programs [39]
2019	Health Sector Integrated Refugee Response Plan (HSIRRP) 2019–2024 [11]
2019	High-level meeting of Ministers in charge of refugees in the Great Lake region on local integration as a durable solution for protracted refugees [40]
2019	Regional Ministers signed the Kampala Declaration that aims to support jobs, livelihoods, and self-reliance for refugees, returnees, and host communities in the IGAD Region [40]

We retrieved 91 records from the literature search (Fig. 1). These included 19 published articles (Pubmed, Scopus, and Ovid Medline) that were finally included based on the study criteria and 71 from gray literature (websites, electronic, library, and online sources. Table 2 outlines key events, and policies related to the integration of refugees into the national health system in Uganda. Over the past decades, several refugee crises took place in Uganda including the influx of Polish refugees during World War II. Initial policies that concerned refugees were restrictive as exemplified by the 1960 Control of Alien Refugees Act (CARA) which attempted to ‘control’ rather than ‘protect’ refugees. During these early periods, Uganda was not a party to the 1951 Convention Relating to Refugees because it was not an independent country. It was not until 1976 that Uganda ratified the Refugee Convention and its 1967 Protocol, and it did so with several reservations.

In the 1990s and early 2000s, the country was faced the influx of refugee. Between 1993 and 1994, over 200,000 Sudanese arrived in the West Nile region because of a civil war in Sudan. The refugees settled in Koboko, then Arua, Moyo, and Adjumani districts [26]. In 2000, over 12,000 refugees fled to Uganda from the DRC. Due to the large and protracted nature of refugee crises in the country and the fulfillment of its obligations to refugees, the GoU in collaboration with the UNHCR developed and adopted the SRS in 1999. The SRS sought to integrate refugee services into existing social and service structures in all refugee hosting districts. Since then, the policies on integration have been evolving. The SRS in 1999 provided and learning for subsequent policies and strategies. This culminated in the country adopting the CRRF set out in the New York Declaration for Refugees and Migrants [41]. At the heart of the CRRF is that refugees should be included in host communities from the beginning. In addition, it outlines strategies that “protect and promote the rights of refugees as enshrined by international law, while easing the pressure on the country of asylum [10].” The CRRF laid the groundwork for the development of several strategies and initiatives that aimed to operationalize the integration of refugees in Uganda.

### Context

The fundamental context that shaped the development of policies for the integration of refugee into the national health system in Uganda was the large and protracted presence of refugees in the country. This ignited the first initial conversations between the government, UNHCR, and other actors about transitioning the management and response to refugee crises in the country from emergency assistance to post-emergency local settlement [42]. This was articulated by several respondents:

*“Because looking at the situation in Sudan, and DRC, you do not see an end in sight to the problems in those countries very soon. So, therefore as a country, we are bound to be with these people staying with us for a very long time and as such if we don’t structure the way we have supporting them we will continue to struggle with the complications of their presence. So, to me, this is the first reason for having a Self-Reliance Integration strategy. The inevitability that we had to do something given the fact that these guys would be around for some time,” (KII-National Policymaker)*

Uganda experienced another refugee influx that required urgent action. On December 15th, 2013, a civil war erupted in South Sudan. Between December 2013 and May 2014, an estimated 74,000 South Sudanese refugees fled to Uganda. By December 2017, there were more than 1 million South Sudanese refugees in Uganda [43]. These events presented several challenges, especially in service delivery in the refugee-hosting districts. This generated urgency for the government to lobby for funding to support the new influx of refugees in the country. According to respondents, Uganda was able to use its open border policy to refugees to boost its international reputation and attract much-needed aid to support the health needs of refugees.

*“In 2014, Uganda experienced a new influx of refugees from South Sudan in our west Nile region. This obviously presented several challenges to the central government and District Local Governments. But let us also not forget that Uganda has been a hospitable country with a more or less open-door policy since the 1950s or 1960s when the first refugees came into the country. This has remained the same other things special may be political will and also the host communities. This has made it a good destination for people refugees from neighboring countries which easily brings in international support.” (KII-National Policymaker)*

Convergence in interests between the UNHCR, the government, and host communities stemmed from three factors. First, UNHCR was faced with funding shortfalls that were happening against a backdrop of increased numbers of refugees and protracted refugee crises in several countries. At the same time, the government of Uganda realized that inadequate funding and unequal allocation of resources to refugees were driving inequities between host and refugee populations (with refugees faring better on several health indicators). From a nationalistic perspective, host communities stood to benefit from improved access to quality services if integration was achieved [42, 44]. This was articulated by one respondent:

*“Funding for refugee protection and assistance has been reducing by the UNHCR and its partners but also the government itself doesn’t have money for all these things. We also saw that more money and support for refugees meant the services within the host districts were not the same as in the host communities, a difference in quality of services exists but by then the gap was actually very big. You would find that the programmes for specific disease interventions were planned and implemented differently in the refuges and the host communities e.g., the malaria control program, vaccination program, and really service delivery were not well streamlined for both the refugee settlements and the host populations and coordinated and implemented differently”. (KII-National policymaker)*

The Ugandan experiment with integration thus took off against this backdrop and continued for many years, with multiple iterations and revisions of policies and strategies. The SRS was the first evidence of an initiative and provided a template that was used to develop policies aimed at the integration of refugees into the national health system. The issue garnered renewed attention in the wake of the Global Compact of Refugees and the New York Declaration for Refugees and Migrants in 2016 [41].

*“The SRS that we implemented as a country was given global recognition for what we were doing at a micro level. But also, the need to have solidarity with countries that are hosting refugees was I think the biggest milestone and change in the thinking that even if you were not hosting refugees don’t feel safe, you should give a hand to those who are already hosting them so in that again refugees should benefit from the social services in the place where they are living, so in this way the milestone every country picked out from”. (KII-National Policymaker)*

Approval and political support for hosting and supporting refugees from the highest offices in the country was key for supporting policies and strategies aimed at the integration of refugee health services into the national health system. This is exemplified by a declaration by the President of Uganda of the country’s determination to continue hosting and supporting refugees. The declaration was immediately followed by several verbal declarations of support by senior GoU officials. As one respondent notes reflecting on the role of the President:

*“He brought all players and said, baaaam (while banging the table). “I am hosting 1.4 million” and he received a lot of support. That is government. Even if the money is coming that is government”. District policy maker quoting the President’s speech at the Refugee Solidarity Summit in Uganda 2017.*

### Content

In 1999, the Office Prime Minister of the GoU and the UNHCR developed the SRS (16). The SRS was the first attempt towards the integration of refugees in the country. The SRS aimed to reduce the reliance of refugees on humanitarian aid by improving the self-reliance and integration of refugee services into District Local Government (DLG) systems in the West Nile refugee-hosting districts. Additionally, it aimed to transition refugee assistance in the country from relief to development programming. It sought sustainable options for protracted refugees in Uganda while ensuring the host community benefited from interventions [45]. The SRS was seen as a means to ensure equity in access to services by refugees and nationals.

In 2003, the SRS was integrated into the wider strategy for Development Assistance to Refugees (DAR) [30]. In Uganda, SRS was institutionalized in the Refugee Act of 2006 and the Refugee Regulations of 2009. The 2006 Act and 2009 Regulations are considered among the most progressive refugee legislations in the world. These regulations grant refugees a wide range of rights including the right to work, education, and access to social services including healthcare [12]. Enshrined in this legal framework is the commitment of the GoU to provide financial and other forms of assistance to refugees. This provided the foundation for the implementation of comprehensive policies and programs aimed at ensuring the well-being of refugees while also contributing to national development.

*“…this comprehensive refugee response was riding on initiatives which we had started way back in Uganda for example from the Self-Reliance Strategy. Now, it gave global recognition for what we were doing at a micro level. The need to have solidarity with countries that are hosting refugees was I think the biggest milestone and change in the thinking that even if you were not hosting refugees don’t feel safe. You should give a hand to those who are already hosting them so in that again refugees should benefit from the social services in the place where they are living, so in this way the milestone every country picked out from”. (KII-National Policymaker)*

Despite the success achieved due to the implementation SRS strategy, respondents indicated that there was a disconnect between the aims of the strategy and systemic and structural factors affecting implementation in the refugee hosting districts. This, respondents note affected the achievements of the aims of the SRS in refugee hosting districts in the country.

*“I will say that Self-reliance practices are not well connected with the prevailing political, economic, and social realities that influence the daily experiences of refugees and hosts. These practices are hindering the very potential for self-reliance. For example, insufficient access to services and the limitations within the settlement policy. We are concerned that these self-reliance measures may undermine the protection and security needs of refugees and the development aspirations of hosts. This obstructs the path to assistance and essential social services. (KII-National Policymaker)*

To improve district-level planning and management in refugee hosting areas, the GoU introduced the Settlement Transformation Agenda (STA) in 2015. The STA operationalizes the National Development Plan II (NDP) (2016–2020) and aims to achieve self-reliance and bring social development to refugees and nationals in refugee-hosting areas. By incorporating the STA into its NDPII, the GoU effectively integrated refugees into the national development planning process. This paved the way for comprehensive responses to address the needs of refugees and nationals. The STA is therefore an entry point for line ministries and development actors into Uganda’s refugee response and reinforces the integration of refugees in the country.

*“We have a Settlement Transformation Agenda, a strategy meant to transform refugee management and to integrate them into the national system. It was developed for full integration and guiding the implementation of refugee programmes but also ensuring that there is no discrimination among refugees and nationals, and seen as a way of improving leadership, governance, and management of refugee health programmes… To a large extent, it is helping on the issue of full integration and ensuring that refugees are provided for in the spirit of humanity”. (KII-National policymaker)*

In September 2016, the UN General Assembly hosted a high-level Summit for refugees and migrants. The objective of the summit was to highlight the challenges of hosting refugees and improve the response of the international community to large movements of refugees and migrants [34]. At the end of the summit, a declaration was signed calling for member states to develop and initiate the application of the CRRF. The CRRF aims to (1) ease pressure on host countries, (2) enhance refugee self-reliance, (3) expand access to third-country solutions (4) support conditions in countries of origin for return in safety and dignity [46]. Uganda signed up and supported the New York Declaration for Migrants and Refugees and agreed to roll out the CRRF in the country. To support the implementation of the STA, the government developed the Refugee and Host Population Empowerment (ReHoPE) strategy [47]. The ReHoPE is a collective humanitarian and development response strategy that sets out to foster a muti-year, multi-sectoral partnership between the GoU, the UN, the World Bank, and humanitarian actors. Among the key objectives is to improve basic service delivery in terms of access, quality, and efficiency.

To translate the CRRF into action, the MoH developed the Health Sector Integrated Refugee Response Plan (HSIRRP) (2019–2024). The HSIRRP is aligned with the National Health Policy (NHP) and the Health Sector Development Plan. The HSIRRP aims to ensure the integration of comprehensive Primary Health Care (PHC) services for refugees into the national and DLG system. The plan prioritizes the use of the decentralized district health system as a means for strengthening health service delivery and coordination mechanisms at the district and sub-district levels. The priority is to provide access to the minimum package of well-coordinated health services for refugees and host communities. Emphasis is placed on preventive and promotive health care for new refugee arrivals at entry points, transit, reception centers, and during their stay in settlements. The healthcare package includes vaccination, nutrition, emergency referrals, lifesaving PHC, surveillance, and management of disease outbreaks. The plan provides a framework for the engagement of DLGs and implementing partners to develop district-specific integrated refugee response plans.

*“As I mentioned, the health sector integrated refugee response plan 2019, is part of the CRRF, coordinated by the OPM and it is aimed at ensuring equitable and well-coordinated access to health services for refugees and the host populations. The plan aligns the refugee health response to the Uganda National Health Policy but also the Health Sector Development Plan which takes up the values of those instruments of the Ministry of Health. The plan gives precedence to the use of the established decentralized health system and provides for a strengthened coordination mechanism at national, uh district and sub-district levels”. (KII-National Policymaker)*

*“We now have an affirmative policy and programmatic environment at national and district levels for improving the provision of health services to refugees and host communities in Uganda. There is a commitment from the government exemplified through the National Development Plan the Health Sector Development Plan and the district-specific Integrated Refugee Response Plans. These key documents have demonstrated health services in the refugee hosting districts and increased attention to the policies has been given to improving health services. (KII – National Policymaker)*

### Process

Faced with the protracted refugee situation in the country the GoU began to explore solutions to address the challenge during the 1990’s. While the SRS was already in place, the actual implementation of integrated services first took place in the year 2000 in Arua district [42]. Until that point, health services for refugees were delivered through parallel structures and systems, which included separate budgets, and administration. This required the reorganization of services to create a unitary health system at the district level that catered to both refugee and host communities [42]. The UNHCR and the OPM held meetings and sensitization events to prepare district administrators and community members for the impending reorganization of the health system. Subsequently, all aspects of refugee health services were handed over to the DLG. The handover process was gradual with facilities transitioning slowly to allow the district health system to adapt to the increased demands and workload. In addition, protocols and guidelines for disease management and response were harmonized. Refresher trainings were organized to ensure that health professionals could respond to the needs of refugees and hosts. Financing was transitioned from the UNHCR to the central government, which in turn transferred funds to the districts. The UNHCR continued to provide budgetary support to the refugee-hosting districts.

Despite it being a well-intentioned approach, implementation faced several challenges. The SRS faced resistance from many district officials. In addition, lack of operational guidance on how to integrate services created confusion that affected its implementation. Another key issue was funding and the extent to which district services, that were already struggling to meet the needs of the host population, could take on the additional burden of providing services to refugees. These and several other issues complicated the implementation of the SRS. An assessment of the SRS in 2004 identified several challenges at the community, district, and national levels that affected its implementation [44].

Following the challenges associated with the implementation of the SRS, the UNHCR intensified its efforts in lobbying the government to ensure that policies on local integration are enacted in Uganda. Together with the government, the UNHCR developed ‘Self-Reliance Strategy–Development Assistance (DAR) for Refugee hosting areas in Uganda. The SRS-DAR aimed to address the drawbacks of the SRS. The SRS-DAR targeted refugee-hosting districts including Adjumani, Arua, Moyo, and Yumbe in the West Nile region. As a result, several policies and legislations were passed that were considered favorable for refugee integration. This included the replacement of CARA with the Refugee Act 2006 and its subsequent expansion and reinforcement by the Refugee Regulations 2010.

As the broader international agenda on the integration of refugees was taking shape, at the national level, Uganda continued its efforts towards integrated services for refugees. While not departing markedly from the earlier policies, the CRRF provided a blueprint for integration and how to translate commitments into action. Since the CRRF was adopted, the country has been implementing integrated services. In some health facilities, implementing partners continue to provide services through contracts awarded by the UNHCR. In others, health services are provided by government facilities alongside implementing partners (both parallel and integrated service delivery models). Gradually, however, health service delivery structures used by implementing partners are being integrated into DLG structures.

### Actors

The SRS was developed jointly by the GoU and UNHCR and implemented by the DLGs. However, the top-down approach to the introduction of the policy and its implementation resulted in resistance from district-level stakeholders [44, 48]. Limited coordination and the lack of common vision among stakeholders were the main challenges in the implementation of the SRS. The need for coordination structures and engagement of relevant institutions and stakeholders was a lesson learned from Uganda’s experience with SRS.

The CRRF relies on an ‘integrated refugee management model’ and is implemented through a multi-sectoral collaboration in the country based on a ‘whole of society’ approach. The rollout of the CRRF is led by the OPM in coordination with UNHCR and the DLGs. A Steering Group and a Secretariat promote coordination and support the implementation of the CRRF. The Steering Group is led by the government and co-chaired by the Minister for Disaster Preparedness, Relief, and Refugees and the Minister of Local Government. The Steering Group consists of 35 members including representatives from the government (ministries, departments, and agencies, DLGs, UN agencies, development partners and donors, refugee representatives, international and national NGOs, and the private sector.

*“The CRRF is a multi-sectoral collaboration implemented by several stakeholders. The government established the Steering Group at national and district levels and is charged with guiding and overseeing the work of the CRRF secretariat and ensuring that it functions effectively. In the years since the establishment of these bodies, it has been advising on the implementation of the CRRF.” (KII-National policymaker)*

Planning for integrated service delivery has been decentralized to the DLGs and sub-district levels. The respective technical departments plan for the various service sectors (education, health, and others). Districts have established planning committees with representation from key stakeholders and technical teams. These committees are charged with developing district-specific integrated plans. One respondent stated:

*“As far as planning is concerned, for us to have a proper comprehensively integrated development plan, we came up with a district planning task force team and this team is comprised of all stakeholders. First and fore most the technical team, the planning committee of the district, then have representatives from the private sector, from the civil society, from the development partners that we have as a result of the refugee interventions, and then of course we have representatives of the refugees by the leader”. (KII-District policymaker)*

To support collaboration and integration between government and humanitarian actors, linkages between refugee sector coordination structures and government-led Sector Working Groups (SWG) were formalized [32]. At the district level, district-level focal points co-chair settlement-related inter-agency coordination meetings. At the settlement level, district government officers have been invited to co-chair SWG meetings.

“*Now one of the biggest changes I have seen as a result of the benefits of efforts for integration is that we have better coordination at national and district levels. At the national level, we have SWGs that are government-designated structures. At the district, the Chairman chars these committees of membership of district local government NGOs and other stakeholders. (KII-District Policymaker)*

## Discussion

Uganda’s experience with implementing integrated health services has important implications for countries grappling with questions of how to provide basic health services to refugees and host populations. Our analysis illustrates that policy learning and evolution were key in the case of Uganda, with policies around integration evolving and improving incrementally, moving the country towards integrated health services. A conducive policy environment was key and a set of legislations, national-level, district-level, and sector-specific plans and budgets that included refugees provided the foundation for implementation. Buy-in at the national level, as exemplified by the involvement of and declarations of support by high-level officials including the president for integration. was coupled with deliberate efforts by the government to mainstream refugee response in local plans and budgets. Examples of the latter included the revision of district planning guidelines to include refugees; the inclusion of refugee population figures in planning and budgeting for the NDP II 2015/2016–2019/2020; and the integration of refugee matters in national sub-working groups. Uganda’s experience illustrates that integration cuts across different sectors and actors at different levels of government. This finding is supported by literature that shows the importance of a multisectoral approach and collaboration in refugee and migrant health services [49, 50].

In addition to political commitment from the government to integration, international support—both financial and technical—was crucial for moving forward the policy agenda on integration of refugees into the national health system. Our analysis shows that the policy process was inherently intertwined with regional, global, and international policies and strategic developments. The influx of South Sudanese refugees in 2014 prompted the inclusion of refugees into the NDP II 2015/2016–2019-2020 and the STA that informs the integration of refugees into the national health system. The adoption of the CRRF and National Action Plans were formulated against the contextual background of the South-Sudanese crisis and subsequent inflow of refugees into the country as well as developments at the global level including the historic New York Declaration [46] on Refugees and Migrants. Like other case studies on refugee response, our study highlights the importance of strong commitment from government and international support towards the integration of refugees into national health systems [51].

Our findings underline the importance of facilitating and institutionalizing multisectoral collaboration to support the policy processes that are key to the integration agenda. The role of key actors was crucial in driving the integration of refugee health services into national health systems. Government ministries, departments, and agencies were actively engaged in agenda-setting. Other important actors played critical roles, especially in ensuring the policies and strategies were inclusive and comprehensive including development and humanitarian partners who had several priorities and concerns about the implications of the policies and frameworks. The importance of a multisectoral approach and collaboration has been highlighted in other settings as well [50]. Indeed, experiences from other settings illustrate the importance of intersectoral and integrated models of refugee integration and response (10). Intersectoral approaches typically involve working across sectors to address the health needs of refugees, while integrated models focus on coordinating health services within the health system to ensure accessibility, cultural appropriateness, and adequate quality of care. Through the years, examples of the latter have included the integrated health services and the SRS in Uganda while one notable example of the latter is Ethiopia’s cross-mandate approach. In Uganda, the aim was to establish mechanisms that would ensure the integration of services for the refugees with those of the nationals (27). The approach in Ethiopia emphasized four guiding principles: collaborative, inter-agency efforts; addressing root causes of assistance; prioritizing need over categorization; and delivering aid through community structures rather than to individuals in camps (51). Integrating refugees into national health systems has several strengths including improved health outcomes and promoting equity and inclusivity (39, 52, 53). However, it is also associated with several weaknesses including straining resources, encountering resistance from host communities, and a lack of national interest in refugees (53). Lessons from past experiences underscore the importance of holistic, rights-based approaches that address the unique health needs of refugees while fostering social cohesion with nationals and collaboration between stakeholders (10).

The findings show that several policies and strategies have been developed and are being implemented to support the integration of refugees into the national health system in Uganda. While these policies have contributed to improving health outcomes and access to services in refugee-hosting districts, available evidence highlights persistent challenges and limitations [52, 53]. Encouragingly, the policies have facilitated increased access to health services, leading to improvements in health outcomes [54]. However, challenges remain, including inadequate health infrastructure, and a shortage of medicines and health workers, particularly in refugee-hosting regions with high population densities [55–58]. Furthermore, socio-economic disparities and competing health needs between refugees and host communities pose challenges to equitable access and delivery of health services [59, 60]. Addressing these challenges will require strengthening policies and strategies to focus on Primary Health Care services [61], ensure equitable access to health services [62], and mainstream refugee health into global, regional, and national policies.

In recent years, integrating refugees into national health systems has gained increasing attention across the globe [63]. Several countries have made initiatives to bridge health service gaps between refugees and nationals. The governments of Jordan and Lebanon, have adopted a comprehensive approach to integrate refugee health into the national health system [64]. Through coordination between health partners Jordan is enhancing the eligibility of refugees to access the national health system. In Iran, registered refugees are now included in the Universal Public Health Insurance [65].

Different approaches have been sought to integrate refugees into national health systems, with variations in models of integration explained by the impact of contextual factors, including political, social, economic, and legal considerations, shaping the trajectory and modality of integration. For instance, countries like Germany and Canada have established comprehensive policies and programs to facilitate the integration of refugees into their national health systems, offering access to a wide range of healthcare services, including primary care, mental health services, and specialized care for vulnerable populations [66]. In contrast, countries in the global south that are relatively more resource-constrained face challenges and struggle to provide adequate health services to populations in refugee settings. South Africa adopted a mainstream model of care for its refugee and migrant population. However, this has been stalled by a complex legislative environment with significant variation in practice. The Refugees Act 1998 entitles refugees to the same basic health services as South African citizens. However, other South African laws, such as the Immigration Act 2002 and the National Health Act 1998, contain sections restricting access to health care for refugees [67]. Ethiopia developed the Refugee Proclamation and adopted the CRRF to inform health service delivery for refugees. However, implementation has been affected by a lack of leadership and coordination [68]. These cases illustrate commonalities, challenges, and opportunities for improving the integration of refugees into national health systems worldwide. These experiences along with Uganda’s experience advancing the integration agenda provide important learnings and insights for countries hoping to find sustainable and rights-based ways to address refugee crises.

## Strength and limitations

This case study focused on the integration of refugee health services into the national health system in Uganda. The study highlights the key contextual features, actors, processes, and drivers that helped advance the agenda of integration in the country. The study interviewed various actors across different government MDAs, civil society, humanitarian agencies, and development partners at national and sub-national levels and applied a framework that takes into consideration the role of political dimensions, role, and interconnected relationship of the key actors in the planning, management, and delivery of health services for refugees and host communities in Uganda.

Several limitations need to be considered. First, our data was collected from district-level policymakers in the West Nile region refugee hosting districts. While this is the major refugee-hosting region in the country, there may be differences in terms of administration in the DLGs, socio-economic factors, coordination and governance, and the overall landscape of health service delivery in other districts. Secondly, temporal constraints must be taken into consideration as the study timeframe (2013 and 2020) might miss recent developments or changes in Uganda’s refugee integration policies and practices, potentially excluding new policies, initiatives, or contextual factors emerging post-study. The first policies were developed more than two decades ago. This might have limited the respondent's recall of the exact events during the early phases of integrating health services for refugees into national health systems. Thirdly, purposive and snowball sampling for semi-structured interviews may introduce selection bias, as more accessible participants may not fully represent all stakeholders involved in efforts aimed at the integration of refugees into national health systems. Lastly, while the study used various data collection methods like document review and key informant interviews, the reliance on these sources may have limitations such as incomplete capture of stakeholders’ perspectives or insights and potential oversight of additional documents or information sources.

## Conclusion

Policies and strategies aimed at the integration of refugee health services into national health systems are critical for shaping coverage, access to, and utilization of health services by refugees and nationals. Our study highlights the complex, dynamic, evolving, and multifaceted nature of the multisectoral health policy process in refugee settings. The findings shed light on the importance of collaboration between stakeholders, mobilization of legal and political frameworks to shape the integration of refugee health services into the national health system, and ensuring that high-level commitments translate to action and development plans at local levels. Findings from this study may help inform learnings among practitioners, policymakers, and researchers about good practices in the implementation of integrated services.

## Supplementary Information

Additional file 1.

Additional file 2.

## Data Availability

Data is available upon reasonable request from the corresponding author Henry Komakech at, hkomakech@musph.ac.ug.

## Abbreviations

CARA: Control of Alien Refugee Act
CCRF: Comprehensive Refugee Response Plan
DAR: Development Assistance for Refugees
DLG: District Local Governments
GoU: Government of Uganda
HSIRRP: Health Sector Integrated Refugee Response Plan
MDA: Ministries Departments and Agencies
MoH: Ministry of Health
NDP NDP: National Development Plan
NGO: Non-Governmental Organizations
NHP: National Health Policy
OPM: Office of the Prime Minister
PHC: Primary Health Care
ReHoPE: Refugee Host Empowerment Project
SRS: Self-Reliance Strategy
STA: Settlement Transformative Agenda
UN: United Nations
UNHCR: United Nations High Commission for Refugees
UNICEF: United Nations Children Emergency Fund
WHO: World Health Organization
