# Supplementary material for: Examining the integration of refugees into the national health system in Uganda: an analysis using the policy triangle framework
Source: Confl Health. 2025 Jan 21;18(Suppl 1):78. doi: 10.1186/s13031-024-00640-2 (PMC11752622; doi:10.1186/s13031-024-00640-2)
Supplement: Supplementary file 1 — Additional file 1. [file 13031_2024_640_MOESM1_ESM.docx]

**Appendix I: Search terms and electronic databases searched**

Database: Ovid MEDLINE(R) and Epub Ahead of Print, In-Process &amp; Other Non-Indexed

Search Strategy:

-----------------------------------------------------------------------------

1 refugee*.ti,ab. (9331)

2 Refugees/ (9373)

3 Uganda/ (11055)

4 uganda.ti,ab. (12717)

5 1 or 2 (12699)

6 3 or 4 (15157)

7 5 and 6 (161)

***************************

**PubMed Search strategy**

"refugees"[MeSH Terms] OR "refugees"[All Fields] OR "refugee"[All Fields]

"Uganda"[MeSH Terms] OR "Uganda"[All Fields]

#1 AND #2

**Scopus search strategy**

TITLE-ABS-KEY ( Uganda ) )  AND  ( ( TITLE-ABS-KEY ( refugee* ) )  OR  ( TITLE-ABS-KEY ( displaced ) ) ) (482 hits)
