# Supplementary material for: Examining the integration of refugees into the national health system in Uganda: an analysis using the policy triangle framework
Source: Confl Health. 2025 Jan 21;18(Suppl 1):78. doi: 10.1186/s13031-024-00640-2 (PMC11752622; doi:10.1186/s13031-024-00640-2)
Supplement: Supplementary file 2 — Additional file 2. [file 13031_2024_640_MOESM2_ESM.docx]

**Appendix 1: Key Informant Interview Guide – Policy/maker/stakeholder**

| **Reference #** | |
| --- | --- |
| Name of interviewer: | Date: |
| Location: | Time: |

1. Can you please briefly describe your job responsibilities and say how long you have been working here?
2. In general, how would you describe the work that this office does?
3. Which populations do you serve or to which office do you report?
4. To what extent do you have direct contact with refugees?
5. What was your professional experience before coming here?
6. In your opinion, what have been the key policy developments since refugees [South Sudanese] began coming here in [2011/2013]?

*Probe: [questions regarding country-specific policies based on desk research]*

1. What do you think are the main reasons these developments occurred?
2. Who or which organizations do you believe had central roles in these policy changes?
3. How would you characterize the role of the MoH in these policy shifts?
4. How do you think these policy developments affected health services provision?
5. How do you think these policy developments have affected front line healthcare providers?
6. In a few sentences, could you describe, in your opinion, what the most important aspects of [country’s] prior experience with refugees are for the current situation?
7. What would you name as the primary sources of financial support for refugee health services in Uganda?
   1. In your view, how effective have these funding systems have been?
   2. How has the refugee situation affected government spending on health care?
   3. How do you think donor funding has affected the quality of healthcare facilities can

provide:

- - 1. To refugees?
    2. To locals?

1. How do you think donor funding has affected the overall quality of care the healthcare system provides? In your view, what are/have been the main challenges in managing healthcare funding? (probe: corruption?)
2. In general, how do you think the care received by Sudanese refugees in Uganda compares to the care local populations receive?
   1. What changes have you observed in health service delivery for refugees and host populations over time?
   2. What kinds of economic and financial factors do you see as affecting care for each population?
   3. In your professional capacity, what kinds of regional differences, if any, are you aware of when it comes to refugees versus local populations’ experience of health care?
   4. What would you say are there local-level conditions or relationships most likely to affect refugees’ healthcare experience?

1. On a scale of one to ten, to what extent do you think the refugees are integrated into the health system in _______? (Uganda).
   - - - 1. Could you explain your answer in a few sentences?
         2. What does it mean for refugees to be “integrated” into the Ugandan healthcare system?
2. If you are at an international conference with someone who has the exact same job as you in [Colombia/Bangladesh/other refugee receiving state]. What advice would you give them, given your experience?
3. Is there anything I should have asked you but did not?

*Thank you very much for your time; we appreciate your participation in this study.*

**KEY INFORMANT INTERVIEW GUIDE - HEALTH MANAGERS/ DISTRICT LEVEL HEALTH MANAGERS**

| **Reference #** | |
| --- | --- |
| Name of interviewer: | Date: |
| Location: | Time: |

1. Can you please briefly describe your job and say how long you have been working here?

How would you describe the work that this facility does?

How would you describe the different populations you serve at this facility? In general, how would you describe your patients?

- - - 1. To what extent do refugees use this facility compared to local populations?

What was your professional experience before coming here?

*For host country providers:* Have you ever experienced displacement in your own life? If yes, *what was your experience of healthcare during your displacement?*

1. What has been your experience dealing/working with refugees versus local populations?
2. What are the main sources of funding for your facility?
3. How has the current situation affected the quality of care your facility is able to provide?
   1. What differences do you see in the quality of care you provide to refugee versus local populations?
   2. In your opinion, what are the main challenges to providing the best possible care to these populations?
   3. Could you describe an example where you were able to overcome a major challenge in this work?
4. Has the refugee situation affected your facility’s capacity to provide health services?
   1. *How have the resources available to you as a provider changed since the refugee situation began?*
   2. *How has your daily routine shifted since the situation began?*

***Probes:*** salaries, professional development, workload, relation to medical com (also include in health manager/district manager protocol)

1. What could decisionmakers do to better support facilities such as yours?
   1. Probe: decisionmakers could be at any level from the facility’s level to the national and international level.

1. On a scale of one to ten, to what extent do you think the refugees are integrated into the health system in _______? (Uganda).
   1. Could you explain your answer in a few sentences?
   2. What does it mean for refugees to be “integrated” into the Ugandan healthcare system?

1. If you are at an international conference with someone who has the exact same job as you in [Colombia/Bangladesh/other refugee receiving state]. What advice would you give them, given your experience?

1. Is there anything that I should have asked you but have not?

*Thank you very much for your time; we appreciate your participation in this study.*
